# Exploring knowledge of autism, its causes and treatment among immigrant and nonimmigrant parents in Somalia\Somaliland

**DOI:** 10.1186/s13034-024-00713-3

**Published:** 2024-02-07

**Authors:** Hodan A. Duale, Abdi Gele

**Affiliations:** 1 Department of Maternal and Child Health, Somali Institute for Health Research (SIHR), Hargeisa, Somaliland; 2 Department of Health Service Research, Norwegian Institute of Public Health, Skøyen, 222, 0213 Oslo, Norway

**Keywords:** Autism, Somali, Immigrants, Knowledge, Parents of children with autism

## Abstract

**Background:**

The prevalence of autism spectrum disorders (ASDs) has increased over the recent years; however, little is known about the experience of parents of children with autism in Africa such as Somalia. The aim of this study is to understand the knowledge on autism of Somali parents of children with autism and their perceptions of causes and treatment of ASD.

**Methods:**

We conducted a qualitative study involving 22 parents of children with autism who lived in Mogadishu and Hargeisa; the two largest cities in Somalia. In-depth interviews were used to collect the data. Of the 22 participants, 9 were returned immigrants and 13 were local people (non-immigrants). Data were analysed using thematic analysis.

**Results:**

The data revealed that most of the parents hold the belief that their children’s autism were caused by the measles vaccine. The findings demonstrated that parents sought diagnosis and treatment care from outside Somalia due to the lack of experience of health providers in the diagnosis and treatment of autism. The data also revealed a lack of knowledge about autism among the public with resultant stigma and discrimination against children with autism and their families.

**Conclusions:**

Efforts to increase public knowledge on autism, its causes and treatments are of paramount importance, while a public health campaign designed to eliminate the stigma subjected to children with autism is necessary to improve the quality of life of children with autism and their caregivers. Finally, to counteract vaccine hesitancy, particularly in response to the measles vaccine, health policy makers should take steps to separate the cooccurrence of the onset of autism symptoms and the provision of the measles vaccine.

## Background

Autism spectrum disorders (ASDs) are life-long neurodevelopmental disorders characterised by changes in shape and size of the brain that affect how people interact with others, communicate and behave [1]. The causes of ASD is not known, but both genetic and environmental factors are known to contribute to the development of ASD. The global prevalence of ASD is reported to be increasing [2], which partially reflects the impact of public health efforts to raise global awareness of ASD. According to a recent WHO report, one in every 100 children have autism [1], ranging from 69\10,000 in France to 151\10,000 [1] in Qatar [3]. However, there is a major gap in what is known about the global burden and global distribution of ASD. In particular, little is known about ASD in Sub-Saharan Africa such as Somalia.

The diagnosis and management of ASD pose a remarkable challenge to communities in Africa due to constrained healthcare resources and limited access to mental health services [4]. Furthermore, cultural factors largely affect how ASD is perceived, which makes it difficult to generalise the available literature to the African context [5]^.^ There are few studies that have examined the prevalence of ASD in some African populations. These studies reported a prevalence of 33.6% and 11.5% among Egyptian and Tunisian children respectively [6, 7]. A study by Oshodi et al. revealed that 34.5% of the children screened in Nigeria had ASD [8],

Global prevalence of autism did not identify any empirical data from Somalia, even though this country has a population of nearly 18 million, 47% of whom are children younger than 14 years [9]. However, a WHO anecdotal estimate shows that 84 per 10,000 children in Somalia have autism [3]. While little is known about autism in Somalia, substantial studies exist among migrants from Somalia in Europe and USA [10–20]. Hewitt et al. reported that the prevalence of ASD was higher in White and Somali children in Minneapolis than in Black and Hispanic children [21]. Similarly, a study in Sweden showed that the prevalence of autism was between four and five times higher in children of Somali background than in those of non-Somali origin [22]. Although there is statistical evidence indicating that Somali refugees and immigrants have high rates of autism spectrum disorder (ASD), Somalis in North America call autism the “Western disease” because there is no word for autism in the Somali language, and because many believe it does not exist in Somalia [23]. Further, a recent study in Sweden showed that Somali immigrants experience higher than average rates of ASD than does the general population. It is also considered a Western disease that only affects Somali children in the diaspora [19]. Similarly, a study in the UK reported that children of Somali descent are six-times (RR 5.99, 95% CI 3.24–10.8, p < 0.001) more likely to be referred for possible autism spectrum than their white or mixed-race peers [24]. So far, studies did not investigate or even postulate reasons for overrepresentation of Somali children in the USA or Europe for ASD. However, gray literature shows that consanguinity is common among Somalis which may put them at increased risk for ASD [16].

Consanguinity is termed as marriage between close blood relations or biological kin mainly second cousins or closer [25]. According to rough estimates, 20% of the global population lives in communities with a preference for consanguineous marriages [26, 27], and it is higher in countries in the Middle East, Africa and South Asia with 65% in Pakistan, followed by India (55%), Saudi Arabia (50%), Afghanistan (40%), Iran (30%), Egypt, and Turkey (20%) [28–31]. Consanguineous marriages were assumed to be a factor of importance in the distribution of aetiologies of developmental disorders such as severe learning disability and ASD [2, 32]. A prior clinical trial evaluated consanguinity as an independent risk factor for the development of ASD by enrolling 500 children with ASD born by parents who are first cousins, second cousins, uncle-niece, and double first cousins. The study found that the ASD increased considerably with consanguineous parents as compared with the controls, with an overall risk factor of 3.22% [32]. Similarly, a study in Lebanon where consanguinity rates are known to be high, reported that consanguinity increases the risk for ASD with an odds ratio of 2.5 [33].

Different cultures vary in their beliefs about the causes of autism. In the United States, 41.6% of study participants mentioned genetics as the cause of autism while 22% mentioned other causes such as pollution, vaccines or viruses [34]. In Kenya, caregiver perceptions about the causes of autism ranged from supernatural beliefs, such as evil spirits, witchcraft and curses, to biomedical causes related to infections, drug abuse, birth complications, malnutrition and hereditary conditions [35]. The parent’s beliefs about causes of autism inform not only their beliefs about their expectations of whether it will be a lifelong disability or whether the child will show recovery, but also the kinds of expectations they hold from the treatments they use with their children. For example, some parents are convinced that if they find the right interventions and use them faithfully, their child will be cured of the disorder [36] Meanwhile, parents who embrace a fatalistic view of their child’s disability (God’s will) are more likely to accept their child’s condition as destiny and have lower expectations of cure or improvements from any treatments used. Although it is important to understand how different cultures perceive the cause, diagnosis and treatment of autism [37], the understanding of autism of Somali families of children with autism in Somalia, and their experiences have not been explored yet. The available knowledge on autism in Africa stresses the pressing need for research that addresses the unique challenges faced by individuals with autism and their families in this context [38]. Parents are the first to recognize signs and symptoms of autism, and their perceptions and beliefs may drive their health behaviours regarding whether to seek formal diagnosis and treatment or choose to wait [39]. The reason for parents to have good knowledge on autism is because parents of children with autism undergo great financial and mental burden and the more uninformed they are, the greater the risk of misdiagnoses and inappropriate treatment. Good knowledge of autism, its causes and treatment will help parents in devising a well-constructed treatment plan, helping release stress, as they will be able to share their concerns with the appropriate healthcare providers. There is a high chance of misdiagnosis or late diagnosis if there is a poor knowledge about the autism, especially among parents of children with autism since they are the first to observe any unusual behaviour compared to other siblings or age groups. Therefore, exploring parents’ perspectives on ASD is of urgent importance as the vast majority of Somalia’s population with autism today is severely overlooked and neglected. The aim of this study is to understand the knowledge on autism of Somali parents of children with autism and their perceptions of causes and treatment of ASD.

## Methods

### Study participants

We conducted 22 in-depth interviews (IDI) with parents of children with autism who attend in one of two main centres in Somalia that provide activities and schooling to children that have diverse developmental disorders. We included parents with children who were diagnosed with autism by a doctor, while we excluded parents with children who had other developmental disorders.

### Recruitment and data collection

Thirteen parents of children with autism were recruited from Mustaqbal centre in Mogadishu, and nine were recruited from Hargeisa Children learning centre: a total of 22 participants (Table 1). Twenty of the parents were mothers, while the remaining were a father and a grandmother. The mothers who reported their age (N = 11) had age range of 22 to 39 years, and 41% (N = 9) had university education. We used a question-guide with semi-structured questions that were constructed to achieve a deeper understanding of the parents’ experiences. We adopted a co-production approach, in which a community organisation (SAHR) was involved in the initiation of the idea, the recruitment of participants, the data collection and the write-up of the results. Co-production research involves collaboration between researchers and end-users of the research [40]. First, we contacted the administration of the two centres who gave us approval to interview parents. Afterwards, parents were informed about the study. Those who showed willingness to participate were contacted through face-to-face meetings. Before starting data collection, information about the study was provided to the participants in different venues based on each participant’s preference. The parents in both Mogadishu and Hargeisa have demonstrated a strong desire to discuss the topic and the situation of their children with data collectors. Parents were motivated by the fact that they wanted to understand more about the condition and to support the research about autism. Data collectors explained about the study objectives, benefit and risks associated with the study to each participant to promote transparency. The interviewers were a public health professional and a medical doctor in Hargeisa and Mogadishu respectively. The interviewers discussed with parents through informal talk about their concerns, after the data collection was completed. The interviews were conducted in Somali which is the native languages of participants and data collectors. The interviews were audio recorded and transcribed verbatim by the interviewers.

**Table 1:** Characteristics of study participants

	Relation (diaspora = D)	Age	Education	No children	Autistic	Age of diagnosis
1	Mother	–	No	5	2	1
2	Mother	29	University	4	1	2
3	Mother	25	Primary	5	1	2.5
4	Mother	31	University	3	1	2
5	Mother	39	primary	1	1	7
6	Mother (D)	38	primary	3	1	2.5
7	Mother	22	University	3	1	2.4
8	Mother (D)	35	Primary		1	2
9	Mother	32	University	4	1	3
10	Grandmother	62	No	2	1	2
11	Mother	28	Primary	2	1	2
12	Mother (D)	38	Secondary	5	1	2
13	Mother (D)	31	University	3	1	3
14	Mother	–	University	6	1	3
15	Mother	–	University	4	1	1,5
16	Mother	–	secondary	7	1	2.1
17	Father (D)	–	University	5	1	1.6
18	Mother (D)	–	University	4	2	2
19	Mother	–	No education	7	2	2
20	Mother (D)	–		3	1	2.8
21	Mother (D)	–	No education	2	1	
22	Mother (D)	38	Primary	4	1	2

*D stands for ‘diaspora’*

### Data analysis

**Fig. 1:**
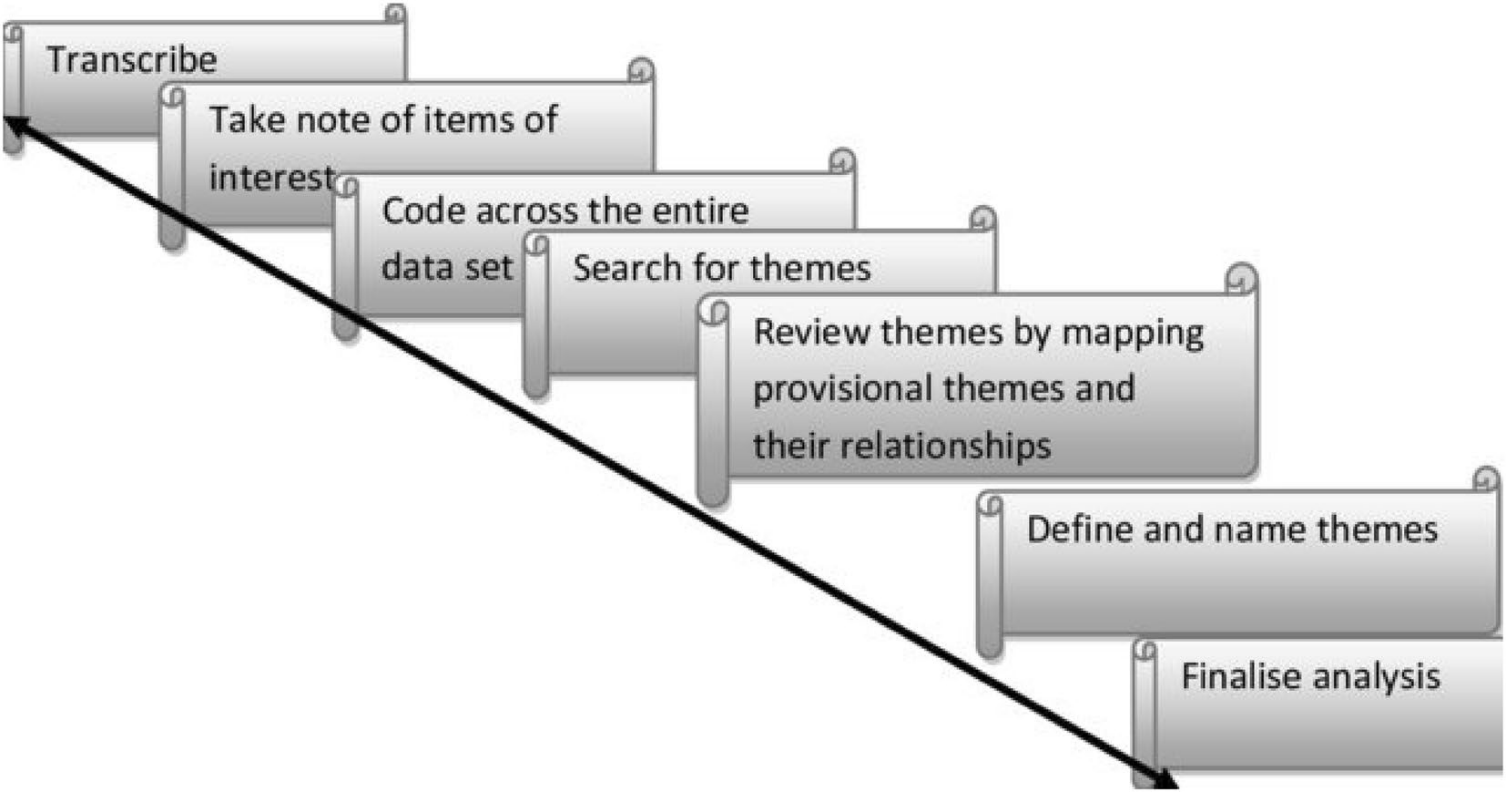
Steps in thematic analysis (Adapted from Braun and Clarke, 2013; 2006)

Data analysis started after the data collection was completed, using a deductive approach. The interviews were first transcribed in Somali, and then translated to English. To validate that each question was transcribed verbatim, the research group reviewed the records several times. To ensure that we accurately reported our participants’ own words and meanings, two authors (HD & AG) listened to the interviews separately and cross-checked the transcriptions. The transcripts were arranged in sequences to organise answers into categories. Subsequently, we began coding and categorising the data based on inter-coder agreements reached by the two authors. Codes were then grouped into different categories, and the categories were subsequently clustered into specific themes using thematic analysis (Fig. 1) [41]. Thematic analysis was associated with resourcefulness and flexibility, as it does not come with fixed instructions [41]. We used NVivo software, version 12, for the analysis of the data.

A wide range of perceptions, ideas and experiences from the diverse in-depth interviews were verified against each other, to help ensure the trustworthiness of the study results. During the analysis, several steps were taken to ensure the qualitative rigor and trustworthiness of the findings. The interview guide was piloted, and subsequently adjusted before it was validated by the authors to ensure that it met the aims of the study. The diversification of information sources (fathers, mothers, grandparents, Somali diaspora, and mainstream Somalis) also ensured the trustworthiness of our results. Our good knowledge of the area, the culture and the language allowed us to minimise biases. Ethical approval was received from the Somali Academy for Health Research and the Somali Institute for Health Research. Prior to data collection, full information about the study was given to participants. Participants were informed that they could withdraw from the study at any time. Verbal consent was obtained from each participant. Questions were designed to minimise the risk of discomfort and the invasion of privacy due to the sensitivity of the study topic.

## Results

The study interviewed 22 parents of children with autism of which nine (41%) were returned diaspora, mostly from Europe and North America, while 59% were non-immigrant Somalis. The participants’ responses were summarised into the following five major themes and sub-themes (Table 2).

**Table 2:** Data structure table

Codes	Themes	Sub-themes
Meeting with a doctor; Diagnosis; medication; seeking treatment abroad; misdiagnosis; doctor do not know; Not heard about autism; Don’t know, never thought it was autism; autism is a foreign disease; training about autism; actions to be taken	Autism is an unfamiliar term	• Knowledge among health providers • Knowledge among participants
When signs appeared; measles vaccine; the cause; children not take the vaccine; suspected causes; depression; breastfeeding; depression; vitamin D, evil eye;	Diverse causes	• Vaccine • Other causes
Treatment; stem cell; health outcome; religious therapy; supernatural; signs of improvement; reliance on Allah	Various treatments	• Medical • Traditional • Fatalistic view
Judgement, blame, discrimination, family, neighbours, bad treatment; harsh words;	Widespread discrimination and judgement	
Recommendations; parent’s needs; awareness; specialists	Unmet need	

## Theme 1. Autism is an unfamiliar term

### Knowledge of autism among health providers

Some of the participants reported that doctors in Somalia do not have enough competence in diagnosing autism. As a result, parents went through different hospitals to obtain authentic diagnosis and treatment for their children. Some families sought expensive and time demanding diagnostic care from outside the country.

*We visited almost all the hospitals in Mogadishu. We visited the Digfer hospital, and they diagnosed my child with epilepsy. Then, we took him to ‘Adan Adde hospital’ which also diagnosed him with epilepsy and prescribed medication. We visited a neurologist, and he told us that the child doesn’t have any neurological illness. Finally, we took the child to Kenya where they told us that the child doesn’t have an epilepsy but autism, so he needs speech therapy *[4]*.*

*…. the schoolteacher called me in for a meeting and told us this child is not only hyperactive, but also he doesn’t have attention, no focus, so he should be taken to a doctor… I took him to a doctor. The doctor said the child has an injury on the head, he might have fallen down and hit against an object, I told him that he never had an injury on the head. Finally, after several misdiagnosis, the child was diagnosed with autism *[19].

*I sought help from a religious leader who first told us of the existence of autism. At that time even our doctors didn’t know autism, they had no idea about the condition. *[15]

### Knowledge of autism among participants

Most of the participants whose child was born in Somalia reported that they have never heard the term ‘autism’ before they get a child with autism. Some participants mentioned that Somalis in the diaspora know this disorder better than they do, therefore if they need information about autism, they ask their relatives in the diaspora.

*The reality is that I don’t even understand what autism is, and it’s my first time that I have heard that a child has a condition called ‘autism’ *[6].

*I didn’t know about autism, but Somalis who live abroad know it, so they share with us about the symptoms of the condition…we used to see children with such challenges, but we did not know that the child had a condition called autism. *[19]*.*

Other participants hold the belief that autism has been prevalent in Somalia, but people did not know it. The participants stated common Somali names which literally means someone who behaves differently, and they said all those names and labels were applied to people with autism.

*We did not know about autism until very recently, but people who had these symptoms used to be everywhere in Somalia. They used to have nicknames such as ‘Sufi’ *etc. *(hyperactive), may be those people had autism, but it has been very recent that we have heard the term ‘autism’* [22].

*This is not a new disease, it existed in our society. I remember children that we used to name them ‘Sufi’, waalow (crazy) or doqon (idiot) *etc*. we were not familiar with the illness…, …some people think that it is more common for Somalis in the diaspora but that is not true *[17]*.*

Many Somalis think that autism is not common in Somalia, but rather among Somali immigrants in the West. However, the participants held the belief that the problem affects diaspora and non-diaspora Somalis equally.

*When the autism centre was established here, people used to say that autism is a foreign disease, and it is not found here (in Somalia). The centre was started with 5 children with autism who came from abroad, but within 3 months the centre shortlisted 88 children born in Somalia, so autism is also common here *[18].

Compared with non-diaspora participants, Somali mothers who came back from Western countries have demonstrated a good understanding of autism, and they often provide advice to their counterparts. They understand symptoms and above all—how to handle a child with autism. Most of the diaspora parents’ children used to go to special education and behavioural intervention centres, while parents received training about autism and how to live with an autistic child. According to parents, having a child with autism creates a lifetime of multidimensional issues and demands for the family, thus, families should develop a new sense of self-organisation as they are coping with the reality of having a child with different demands and needs.

*Families need awareness of how to deal with children with autism…when my child was diagnosed with autism, we received education about how to deal with autistic children, so that the child becomes happy and the whole family becomes happy…the whole family life should be adjusted to the child with autism… *[18]*.*

*If the child has autism, he\she should not be allowed to continue doing the wrong things such as urinating on the fence. If you allow him, it will take years to reverse this behaviour…let him cry at the beginning, he will stop crying and will understand that this behaviour is not acceptable …eventually he will be fine…(P20).*

## Theme 2. Diverse causes

Participants raised a number of issues that they think were behind the health condition of their children. They narrated about events that occurred shortly before they spotted that the child was behaving differently. The issues raised as causes of autism included measles vaccine, prolonged labour, tuberculosis, evil eye and interrupted breastfeeding. The understanding of parents’ perceived causes of autism is important because it may determine participants’ help seeking behaviour.

### Vaccine

Nearly half of the study participants think that vaccines are the major cause of their children’s autism. The most mentioned vaccine was the measles vaccine. According to participants, the timing of the vaccine, which is the 10th month, co-occurred with the time that the child reversed his\her normal behaviour to unusual behaviour including lack of attention, speech problem, no focus etc. As a result, most of the participants reported that they did not vaccinate the younger children with the measles vaccine. Most participants presented stories about the fact that their child did not have autism prior to the measles vaccination. Here are the participants own words:

*His problem started at 10th month when he got the measles vaccine. He developed skin-allergy, which advanced to skin infection and then autism. Now, I didn’t give the measles vaccine to my younger children. Recently, there was a campaign warning about imminent outbreak of measles, but I decided not to give vaccine to other children—‘let the measles come’!* [15].

*My conclusion is that the measles vaccine was the cause of my child’s autism. He was given at the age of 9th month, and he developed the problem from that day onwards. It is not only me, but many Somalis agree that measles vaccine and autism are related. I didn’t give the vaccine to the younger two children, and I always urge people not to take the measles vaccine (P17).*

A participant who returned from Europe also shared the concern surrounding vaccines with non-diaspora counterparts. The participant demonstrated good knowledge of symptoms, treatments and other aspects of autism, yet, she had a strong opinion associating vaccines with autism.

*I believe vaccines are the cause of autism. My child was fine before the vaccine. My child developed a severe fever immediately after the vaccine. The doctor prescribed a painkiller. The fever didn’t subside, then he was admitted to the hospital. After a month, the child’s behaviour changed, he became lonely, and started running away from people. That is why I believe the vaccine is the main cause *[20]*.*

### Other causes

Parents cited a range of other explanations to account for their son's or daughter's disability, including birth trauma and illness during pregnancy, tuberculosis, breastfeeding, and vitamin D deficiency or depression of the mother during pregnancy. These biomedical attributes were mentioned by four participants.

*There are issues that I suspected to have caused my child’s autism, such as the prolonged labour that I experienced when giving birth to the child, and tuberculosis that infected the child at an early age (2.5 years). (P2).*

*There was a time I travelled and left the child at home. He was 6 months old and breastfeeding. Perhaps he developed this condition because of the interrupted breastfeeding* [7]

*I think it was because of the depression that I suffered when his father died in a terrorist explosion at that time. (P5)*

*I think autism is caused by a combination of factors including vitamin D deficiency of the mother. I had severe vitamin D deficiency when I was pregnant with this child, which may be the cause 18).*

Some parents also expressed beliefs that did not conform to biomedical explanations, but rather drew on magical or religious beliefs. As a result, people sought care from traditional healers, mainly through faith-based therapy including prayers, drinking holy water etc.

*A woman who is my neighbour came to me for a visit and said, ‘your child is beautiful’. After one week, the boy displayed symptoms of autism, he became hyperactive, running, hitting walls, hitting other kids *etc*. *[19]*.*

## Theme 3. Various treatments

### Biomedical treatment

Participants sought treatment for the condition of their child. At least four participants mentioned that stem cell treatment which is found in India were used. Others sought treatment for comorbidities. Some participants mentioned that the child made slight improvement after stem-cell treatment while others think that it didn’t help.

*I took the child to India, they told me that the child has autism, they also treat him with stem cells. I saw the same improvements. Previously, she could not take off her shoes when we came home from outside, but with stem-cell therapy combined with training, she began to take off her shoes. (P 2).*

*My friends took their children to India for stem cell treatment, some children have improved from that therapy, while others did not *[17].

*He was taken to Turkey and was diagnosed with autism. The child later experienced epilepsy when he was 4 years old… they couldn’t control the epilepsy in Hargeisa. We took him back to Turkey, and he received treatment for epilepsy. After the epilepsy treatment, he became more relaxed than before *[15]*.*

### Traditional treatment

Almost all participants sought traditional treatment for autism. The most sought was faith-based treatment in the form of the Quran. Being Muslim, participants strongly believe that the Quran treats everything, thus regardless of whether they see improvement, they seem to have hope that their child will recover 1 day from the Quran therapy.

*I visited different religious leaders, I read the Quran on him many times, we believe that the Quran can treat everything *[6].

*I took him to a religious leader for help and he said that the child has an evil eye. He read the Quran on him, and then he improved slightly. I also took him to Rusheeye (a well-known traditional healer). He said the child has an evil eye and I should treat him with the Quran…he treated the child with holy water splashed on him. Then, some obvious physical symptoms disappeared after the Quran. When I came back to our neighbourhood, everyone saw the improvement of the child *[19]*.*

However, others sought care from traditional healers who often convince parents that their child has been bewitched or that the child has Jinn in him that can be removed through the Quran. The Quran reading is accompanied by beating the patient with a stick or hand and splashing holy water on the body of the patient to chase away the jinn from the body. Other healers use other practices such as removing some teeth from the child.

*I took him to a religious leader; he told me that the child has jinni in his body. He provided diverse religious treatment, that is, when my child started to talk* [15]

*My mother-in-law told me that the condition is called shaasho, and there is a traditional way of treating it such as burning and smoking certain herbs, hair or nails. After doing so, the child’s condition slightly improved. I took him to different doctors, and they told me that he is fine. At the age of 2 years, the child could not stand or walk. My mother-in-law said that the child has ‘ilka-dacawo\dawaco’ (fox teeth), then we took him to a traditional healer that removed the child’s four primary cuspids. The child also received skin burns for treatment. For that, the child started walking and after 3 years he started talking but with some difficulties such as murmuring *[19]*.*

### Fatalistic view of treatment

The vast majority of the study participants demonstrated full acceptance of the child’s condition but maintained the hope that their child will recover from this condition. This acceptance is enforced by the prevalent fatalistic view that the child was created by God with this condition. This is characterised by a constructive reliance on faith. Some parents believed that God could heal their child even when doctors had pronounced that their child’s condition has no treatment.

*I think my child only needs the Quran and I will pay charity for him, because our prophet told us to give charity, so all problems will go. He is fine now, sometimes he refuses to eat but other than that he is fine, Alhamdulillah *[10]*.*

*People were saying that autism has no treatment, but Allah has treatment for every disease. (P17)*

*Allah knows my child’s treatment *[6].

## Theme 4. Widespread discrimination and judgement

Perceived negative attitudes toward autism in the Somali community meant that many families experienced prejudice and discrimination. Participants stated that even relatives find it difficult to maintain contact with parents of children with autism given the fact that, if they visit the family, they feel uncomfortable with the child’s hyperactive behaviour. Some participants also reported that parents in the neighbourhoods do not allow their children to play with autistic children for fear that they will ''catch'' the disability. Further, parents reported that they hear derisive slang expressions that are referred to their children with autism or to the parents themselves.

*My family such as my husband and my mother always say to me that the reason why she is behaving this way is because I spoiled her. She wasn’t communicating with others until she was 4 years old. (P2).*

*My son meets discrimination from the people because they always say “he is the boy who can’t speak, or she has a boy who can’t speak or even they say she has a crazy boy. When he goes out to play with other children, they say similar words to him *[7]*.*

*Our people are not kind to people with disabilities. If you have weakness—whether mental or physical—they name it after you. They call him the ‘crazy boy’. Others blame me for the condition of the child *[15]*.*

*If the child has autism, the mother will have the name ‘mother with the sick child’…if the mother becomes pregnant again, everyone is saying, ooh I hope you do not have another child that has the same problem *[18]*.*

*Our people call this disease ‘Waalli’ (crazy person) … people treat my children in different ways, but 70% of the people do not treat people with disabilities well. It is because of ignorance and lack of understanding…people are rude to children with autism…they call them crazy, idiot *etc.*…(P20).*

## Theme 5. Unmet needs

According to parents, general awareness of the public regarding autism and other disorders, to eliminate discrimination against people with disabilities is of paramount importance in Somalia. Some participants noted the significant need for special schools with competencies in diverse intervention for children with developmental disorders such as autism. Many parents who have children with autism keep their children indoors, because they don’t know where they can receive help or care. Further, parents of children with autism reported that they need information about autism and where to seek care for it. They also highlighted the necessity for specialised health care providers and teachers with competence in special education.

*People should receive awareness about the condition so that they understand about this condition and seek speech-therapy and other behavioural interventions *[22].

*Religious leaders and government institutions should initiate awareness about autism so that people with disabilities and their families are respected by the public. Moreover, we need doctors who can diagnose and treat autism and teachers who can understand children with autism *[15]*.*

*We need people who have knowledge of this disease and centres that provide care for children with autism…people need awareness about this disease because there are people who have children with autism who don’t know what to do and where to take the child for care. I met a woman who has two children with autism but who doesn’t know what to do, she even doesn’t know the condition autism, she only knows that her boys have problems *[17]*.*

## Discussion

To our knowledge this is the first study to explore the experiences of families of children with autism in Somalia, and it is novel in demonstrating parents’ knowledge of autism as well as the care provided to children with autism in Somalia.

As parents are the first to recognize signs and symptoms of autism, research shows that parents’ knowledge and awareness of autism will affect the diagnosis, treatment, and prognosis of children with ASD [42]. Our study shows that most of the participants have never heard of the term ‘autism’ prior to having a child with autism, which concords with prior study in the UK, that Somali parents of children with autism felt there is a lack of understanding of autism in the Somali community due to there being “no word for autism in the Somali language” [17]. While participants in our study have met people with autism-like symptoms before their autistic son or daughter was born, they didn’t know the name of the illness, its causes, diagnosis and treatment. The diaspora members, however, who participated in our study have shown much better knowledge on how to care for children with autism. This finding is in-line with prior findings that parents whose children are already enrolled in schools and therapy centres, are more aware about the intricacies of autism [39]. However, some diaspora members shared some of the myths, such as measles vaccine causes autism with their counterparts in Somalia, which accords with findings of studies among Somalis in the USA [18] and Scandinavia [19]. The poor knowledge of autism among study participants indicates that family members of children with autism undergo great financial and mental burden from seeking therapies that cure autism and the more uninformed they are, the greater the risk of moving from a costly ineffective therapy to another.

The study found that none of the Somali parents in this study believed that autism is a genetic disease or partially caused by genetics, while parents hold the perception that their children *acquired* autism as opposed to being born with it. In-line with the findings of a prior study, our study participants opposed the idea that their children suffer from regressive autism, in which children reach developmental milestones (such as language acquisition) before starting to regress at an early age [43]. As a result, many parents believed vaccines caused their children’s autism and they were certain that their children displayed no symptoms until the day they received the measles vaccine, rejecting the assertion that autism was present from birth. This finding concords with those of studies among Somalis in Europe and the USA [18, 19]. The hypothesis that the measles vaccine can cause regressive *autism* has been tested in Japan using data on 904 patients with ASD, and the study revealed no significant difference in the incidence of regression between MMR-immunised children and non-immunized children [44]. While the cause of autism has yet to be established, it is not unusual that parents of children with autism develop their own perceptions of the potential causes of autism. Somali parents’ explanations for autism may capture their experiences in lack of access to diagnostic care, behavioural intervention, or any other form of care for their children with autism.

The parent’s perception that autism is caused by the measles vaccine is counterproductive to global efforts to eradicate measles, particularly in measles endemic countries such as Somalia. Low measles vaccination coverage leads to recurrent measles outbreaks [45]. Somalia has experienced frequent measles outbreaks in recent years with the latest being 2022. WHO and UNICEF estimated that Somalia’s first dose of measles immunisation coverage was 46% in 2020 [46], despite WHO’s efforts to ensure routine immunisation coverage with MVC1 and MVC2 of at least 95%. Further, the Somali diaspora has been the source of most measles outbreaks in some Western countries for the last decade. Approximately 18 measles cases that were reported in Norway, majority were people of Somali descent [47]. Similarly, an unvaccinated US-born Somali child was infected after visiting Kenya in 2011, leading to more than 20 measles cases in Minnesota. The most extensive US measles outbreak occurred in Minnesota in 2017; 75 cases were reported, over 90% were unvaccinated, and more than 80% were from the Somali community in Minnesota given the fact that measles vaccination coverage in US-born Somali children was 42% at that time [48, 49]. The measles vaccine hesitancy due to the prevailing perception that it causes autism, demands public health measures designed to challenge that perception. One of these measures could be the postponement of the first dose of the vaccine to divert the co-occurrence of the timing of the first dose and the appearance of the earliest symptoms of autism as believed by parents.

Further, many parents believe that their children can fully recover from autism with proper therapies, compelling them to seek biomedical and traditional therapies for the condition of their children. Despite parents seeking diverse treatments such as stem-cell treatment, they reported that their child didn’t recover. In contrast to our study, disability is often viewed as a punishment or curse in several African cultures, leading families to avoid seeking treatment for their child [50]. The treatment approaches of participants may be informed by the fact that Somalis often believe that mental illness stems from multiple sources and cannot be resolved by a single treatment, epitomised by the Somali proverb: “A sick man is advised by a hundred” [20]. The alternative therapy sought most often by our study participants was religion-based therapy in the form of the Quran. This finding concords with a prior finding stating that religion is a highly valued source of healing among Somali communities [17]. It is usual for parents of children with autism to seek out alternative therapies such as religion-based therapy, partially because the existing medical establishment fails to give them hope [51]. In a survey of 1006 randomly selected members of the general public in the US, 68.3% stated that their religious beliefs would guide their medical decisions if they were in critical medical situations, and ‘57.4% believed that God could heal a patient even if doctors had pronounced further medical efforts to be futile’ [52]. Our finding is also in-line with previous research reporting that Somalis turn toward religion-based therapies for autism for many of the same reasons as anyone else [20].

The study shows that health providers in Somalia do not have good experience in diagnosing and caring for autism patients, which may contribute to underdiagnosis and misdiagnosis of autism. This emulates the reality in Africa where there is a severe shortage of trained mental health professionals for autism, and those available are not often trained in diagnosing ASD [53]. According to the WHO, “one in every three Somalis is affected by a challenge related to their mental health, but there are only a few health facilities offering mental health services and there are only 3 psychiatrists and 25 trained nurses dealing with mental health throughout Somalia” [53]. Therefore, parents of children with autism in Somalia sought expensive diagnosis and treatment of autism outside the country, potentially impoverishing the already poor communities living in one of the poorest countries in the world. A prior study stated that ASD imposes a considerable financial burden on families and caregivers in Africa, leading to increased stress levels [54]. Efforts should be made by health policy makers, international NGOs, and academic institutions in Somalia to train mental health professionals with competence in diagnosing and treating ASD.

The study participants reported widespread discrimination, stigma, labelling, and judgement subjected to children with autism and their parents. Almost all the study participants, be it diaspora or non-diaspora, have experienced discrimination. A prior study in the UK showed that the discrimination, stigma and judgements subjected to children with autism and their families are associated with a lack of awareness of autism as a condition among Somalis, and with widespread prejudice against mental illness and disabilities [55]. Discrimination and stigma refer to people’s experience of unfair treatment, while ‘felt stigma’ refers to the shame and expectation of discrimination that prevents people from talking about their experiences and seeking help [56]. Most parents in our study reported that they were blamed by their neighbours and family members for their child’s behavioural problems. This finding concords with a study in Egypt where mothers of children with autism felt socially isolated and embarrassed about their child’s behaviour in public, with subsequent decision of keeping children at home in case of a social event such as birthday or wedding etc. to avoid discrimination and stigma [57].This finding is in-line with the findings of a study in Hong Kong where all the interviewed parents reported fear that their child’s disability would result in discrimination and that the family would be judged in accordance with the traditional belief that a child’s behaviour and success directly reflect on the parents [58]. This finding is also consistent with a body of research indicating that people unfamiliar with autism draw on explanatory models in which the behavioural manifestations of autism relate to parents’ moral failings or poor discipline [55, 59].

Finally, parents expressed a lack of access to services due to the absence of qualified doctors for the diagnosis and treatment of autism and the scarcity of schools for special needs that can provide education and lifelong skills to children with autism in Somalia. This reflects that the diverse needs of children with autism in Somalia aren’t being met. In the USA, 74% of students with autism graduate with a diploma, versus 86% of all students, while nearly 60% of people with autism are employed after receiving vocational rehabilitation services [60]. The success in the US suggests that, with increased public awareness about autism and training competent health providers and teachers, children with autism in Somalia can achieve their own potential like everyone else.

This study has both strengths and limitations. We chose to recruit a relatively small sample, which reflected our exploratory aim of obtaining insight into these seldom heard parents and enabling in-depth analysis. We interviewed parents of children with autism, but not health providers. Thus, important information might be missing regarding the challenges faced by health providers in diagnosing and treating people with autism. The strength of our study is that the discussions and interviews took place in a very open atmosphere, and answers were not imposed through predetermined questions. Most of the views and opinions were repeatedly expressed among different individuals.

## Conclusion

Efforts to increase public knowledge on autism and its diagnosis, symptoms, and treatment are of paramount importance. A public health campaign designed to eliminate the stigma experienced by children with autism is necessary to improve the quality of life of children with autism and their caregivers. An advocacy campaign involving media, religious institutions, schools, mosques, and community outreach programs may effectively achieve this goal. To improve autism care in Somalia, the governments and other NGOs that fund healthcare in Somalia should prioritise funding for short- and long-term plans for training professionals and parents in evidence-based behavioural treatments, speech and language therapies for the management of ASD in Somalia. Finally, to counteract vaccine hesitancy, particularly in response to the measles vaccine, health policy makers should take steps to separate the co-occurrence of the onset of autism symptoms and the provision of the measles vaccine.

## Data Availability

Not applicable (the qualitative data that we collected were included in the manuscript).
